# Supplementary material for: Biomarkers for Inner Ear Disorders: Scoping Review on the Role of Biomarkers in Hearing and Balance Disorders
Source: Diagnostics (Basel). 2020 Dec 29;11(1):42. doi: 10.3390/diagnostics11010042 (PMC7824431; doi:10.3390/diagnostics11010042)
Supplement: Supplementary file 1 [file diagnostics-11-00042-s001.pdf]

Supplementary data:
Detailed search strategies

Ovid MEDLINE(R) and Epub Ahead of Print, In-Process & Other Non-Indexed Citations and Daily: 1946 to January 10, 2019

#	Search Statement	Results
1	exp *Biomarkers/ [mp=title, abstract, original title, name of substance word, subject heading word, floating sub-heading word, keyword heading word, protocol supplementary concept word, rare disease supplementary concept word, unique identifier, synonyms]	23,3542
2	biomarker*.ti,ab.	206,447
3	bio marker*.ti,ab.	585
4	Otolin-1.mp.	17
5	*Exome/	1,956
6	exp *Connexins/	7,432
7	connexin*.ti,ab.	11,271
8	(exome or exomes).ti,ab. [mp=title, abstract, original title, name of substance word, subject heading word, floating sub-heading word, keyword heading word, protocol supplementary concept word, rare disease supplementary concept word, unique identifier, synonyms]	13,723
9	pendrin.ti,ab.	467
10	Pericytes.ti,ab. or exp *PERICYTES/	5,749
11	*Melanocytes/	5,424
12	melanocytes.ti,ab.	11,168
13	(1 or 2 or 3 or 4 or 5 or 6 or 7 or 8 or 9 or 10 or 11 or 12) and (immunolog* or neuro*).mp. [mp=title, abstract, original title, name of substance word, subject heading word, floating sub-heading word, keyword heading word, protocol supplementary concept word, rare disease supplementary concept word, unique identifier, synonyms]	114,750
14	exp Ear, Inner/	51,977
15	otoconia.ti,ab. or exp *Otolithic Membrane/	1,543
16	exp *cochlea/ or exp Mandibulofacial Dysostosis/	21,300
17	cochlea*.ti,ab.	39,790
18	vestibul*.ti,ab.	42,431
19	vertigo.ti,ab.	12,666
20	sensory neural hearing loss.mp. [mp=title, abstract, original title, name of substance word, subject heading word, floating sub-heading word, keyword heading word, protocol supplementary concept word, rare disease supplementary concept word, unique identifier, synonyms]	168
21	inner ear*.mp.	15,103

22	Autoimmune Inner Ear Disease.mp. or AIED.ti. or Bilateral Vestibular Hypofunction.mp. or CANVAS Syndrome.mp. or Central Vestibular Disorders.mp. or Enlarged Vestibular Aqueduct.mp. or General Vestibulopathy.mp. or Labyrinthitis.mp. or Vestibular Neuritis.mp. or "Mal de Debarquement".mp. or Migraine Associated Vertigo.mp. or Vestibular Migraine.mp. or Neurotoxic Vestibulopathy.mp. or Ototoxicity.mp. or Pediatric Vestibular Disorder*.mp. or Perilymph Fistula.mp. or Persistent Postural Perceptual Dizziness.mp. or Secondary Endolymphatic Hydrops.mp. or Superior Canal Dehiscence.mp. or Tinnitus.mp. or Vestibular Hyperacusis.mp. or Sudden hearing loss.mp. or Nonsyndromic hearing loss.mp. or Auditory neuropathy.mp. or chronic subjective dizziness.mp. [mp=title, abstract, original title, name of substance word, subject heading word, floating sub-heading word, keyword heading word, protocol supplementary concept word, rare disease supplementary concept word, unique identifier, synonyms]	21,169
23	(usher syndrome* or otof mutation* or waardenburg syndrome* or treacher collin or pendred or neurofibromatosis or accoustic schwannoma or noise induced hearing loss or alport or norrie syndrome* or stickler syndrome* or marshall syndrome* or wolfram syndrome* or gjb2 or dfnb4 or branchiootorenal syndrome*).ti,ab. and (ear or ears or cochlea or vestibule or hearing or hear).mp. [mp=title, abstract, original title, name of substance word, subject heading word, floating sub-heading word, keyword heading word, protocol supplementary concept word, rare disease supplementary concept word, unique identifier, synonyms]	5,453
24	or/14-23	125,535
25	13 and 24	403
26	limit 25 to animals	158
27	limit 26 to humans	59
28	(25 not 26) or 27	304

Embase: 1974 to 2019 January 10, 2019

#	Search Statement	Results
1	exp *biological markers/ [mp=title, abstract, heading word, drug trade name, original title, device manufacturer, drug manufacturer, device trade name, keyword, floating subheading word, candidate term word]	67,053
2	biomarker*.ti,ab.	324,153
3	bio marker*.ti,ab.	1,612
4	Otolin-1.mp.	21
5	*Exome/	8
6	exp *gap junction protein/	3,422
7	connexin*.ti,ab.	14,535
8	(exome or exomes).ti,ab. [mp=title, abstract, heading word, drug trade name, original title, device manufacturer, drug manufacturer, device trade name, keyword, floating subheading word, candidate term word]	26,039

9	pendrin.ti,ab.	607
10	Pericytes.ti,ab. or exp *pericyte/	7,650
11	*melanocytes/	5,068
12	melanocytes.ti,ab.	15,342
13	(1 or 2 or 3 or 4 or 5 or 6 or 7 or 8 or 9 or 10 or 11 or 12) and (immunolog* or neuro*).mp. [mp=title, abstract, heading word, drug trade name, original title, device manufacturer, drug manufacturer, device trade name, keyword, floating subheading word, candidate term word]	66,630
14	exp *inner ear/	25,247
15	otoconia.ti,ab.	497
16	Mandibulofacial Dysostosis.mp.	1,598
17	cochlea*.ti,ab.	43,137
18	vestibul*.ti,ab.	47,150
19	vertigo.ti,ab.	16,733
20	hearing loss.ti,ab. [mp=title, abstract, heading word, drug trade name, original title, device manufacturer, drug manufacturer, device trade name, keyword, floating subheading word, candidate term word]	49,487
21	inner ear*.mp.	25,198
22	(Autoimmune Inner Ear Disease or AIED or Bilateral Vestibular Hypofunction or CANVAS Syndrome or Central Vestibular Disorders or Enlarged Vestibular Aqueduct or General Vestibulopathy or Labyrinthitis or Vestibular Neuritis or Mal de Debarquement or Migraine Associated Vertigo or Vestibular Migraine or Neurotoxic Vestibulopathy or Ototoxicity or Pediatric Vestibular Disorder* or Perilymph Fistula or Persistent Postural Perceptual Dizziness or Secondary Endolymphatic Hydrops or Superior Canal Dehiscence or Tinnitus or Vestibular Hyperacusis or Auditory neuropathy or chronic subjective dizziness).ti,ab.	21,655
23	((usher syndrome* or otof mutation* or waardenburg syndrome* or treacher collin or pendred or neurofibromatosis or accoustic schwannoma or alport or norrie syndrome* or stickler syndrome* or marshall syndrome* or wolfram syndrome* or gjb2 or dfnb4 or branchiootorenal syndrome*) and (ear or ears or cochlea or vestibule or hearing or hear)).ti,ab. [mp=title, abstract, heading word, drug trade name, original title, device manufacturer, drug manufacturer, device trade name, keyword, floating subheading word, candidate term word]	3,642
24	or/14-23	156,217
25	13 and 24	657
26	limit 25 to animals	117
27	limit 26 to human	0
28	25 not 26	540
29	remove duplicates from 28	524
30	from 29 keep 1-524	524

CINAHL: January 22, 2019

#	Search Statement	Results
1	(MM “Biological Markers”) OR (MH “Antibodies, Antineutrophil Cytoplasmic”) OR (MM “Genetic Markers”)	13, 692
2	TI biomarker* or “biological marker*” or “bio marker”	14,655
3	AB biomarker* or “biological marker*” or “bio marker*”	32,503
4	AB otolin-1	3
5	TX exome	2,404
6	TX connexins	1,097
7	(MM “Connexins”)	11
8	TX pendrin	91
9	TI pericytes	71
10	AB pericytes	333
11	TI melanocytes	57
12	AB melanocytes	548
13	(S1 OR S2 OR S3 OR S4 OR S5 OR S7 OR S8 OR S9 OR S10 OR S11 OR S12	51,996
14	“immunolog*”	73,450
15	neurolog*	67,668
16	S14 OR S15	139,779
17	S13 AND S16	3,522
18	(MM “Ear, Inner+”)	3,804
19	TI “inner ear” or (cochlea* not implant*) or vestibul* or otoconia otolithic or vertigo or “sensory neural hearing loss” or hear or hearing	60,216
20	AB “inner ear” or (cochlea* not implant*) or vestibul* or otoconia otolithic or vertigo or “sensory neural hearing loss” or hear or hearing	60,268
21	TI (“usher syndrome*” or “otof mutation*” or “Waardenburg syndrome*” or “treacher collin” or pendred or neurofibromatosis or “acoustic schwannoma” or alport or “norrie syndrome*” or stickler syndrome*” or “wolfram syndrome*” or gib2 or dgnb4 or “branchiootorenal syndrome*”) AND TX (ear or ears or cochleas or vestibule or hearing of hear)	327
22	S18 OR S19 OR S20 OR S21	60,883
23	S17 AND S22	30
24	Hearing loss or deafness or hearing impairment or deaf or hard of hearing	27,564
25	S1 OR S2 ORS3	47,633
26	(S1 OR S2 OR S3) AND (S24 AND S25)	71
27	S23 OR S26 (not (implant or implants))	46

BIOSIS Previews on Web of Science platform: January 11, 2019

#	Search Statement	Results
1	TITLE:(biomarker or bio marker*" or "biological marker*") AND TOPIC:("inner ear*" or hear or hearing or deaf * or cochlea* or vestibule* or vertigo or tinnitus) Refined by: MAJOR CONCEPTS: (HUMAN MEDICINE MEDICAL SCIENCES) Timespan: All years.	37

CA Plus on the Scifinder Platform: January 11, 2019

#	Search Statement	Results
1	"biomarkers and inner ear"	1166
2	Refine "human"	751
3	Refine "CAPLUS"	302

Web of Science Indexes (SCI-EXPANDED, SSCI, A&HCI, CPCI-S, CPCI-SSH, BKCI-S, BKCI-SSH, ESCI, CCR-EXPANDED, IC), Timespan, All years: January 11, 2019

#	Search Statement	Results
1	TITLE:(biomarker* or "bio marker*" or "biological marker*") *AND* TITLE:("inner ear*" or hear or hearing or deaf* or cochlea* or vestibule* or vertigo or tinnitus) *NOT* TOPIC:(animal* or dog or dogs or songbird* or turkey or turkeys or chicken* or duck or ducks or geese or goose or rat or rats or mice or cat or castor pig or pigs or piglet* or puppy or puppies or horse or horses or rabbit* or canine or bovine or equine or rodent* or porcine or caw or cows or calves or calf or pony or goat or goats or sheep)	25

Cochrane Library Trials: January 11, 2019

#	Search Statement	Results
1	"biomarker* or "bio marker*" or "biological marker*" in Record Title AND ("inner ear*" or hear or hearing or deaf* or cochlea* or vestibule* or vertigo or tinnitus) in Title Abstract Keyword - (Word variations have been searched)"	12

PROSPERO: February 22, 2019

#	Search Statement	Results
1	Biomarker* or "biomarker*" or "biological marker*"	13,57
2	"inner ear" or vertigo or cochlea or vestibule or hearing or hear	566
3	#1 AND #2	9
4	animal* or mouse or mice or dog or dogs or cat or cars or rabbit or rabbits or bunny bunnies or pig or pigs or swine or porcine or equine or bovine or rodent* or feline or canine or sheep or lamb or lambs or cow or cows or calves or kitten or puppy or puppies	5509
5	#3 NOT #4	8

Proquest Dissertations and Theses Global: February 22, 2019

#	Search Statement	Results
1	noft("bio marker*" OR biomarker* OR "biological marker*" OR exome OR connexin* OR otolin-1 OR exomes OR pendrin OR pericytes OR melanocytes) AND noft("inner ear" OR cochlea OR vestibul* OR vertigo OR otoconia OR otolithic OR hearing OR deaf OR deafness OR aied OR dizziness) NOT noft(animal* or mouse or mice or dog or dogs or cat or cats or rabbit or rabbits or bunny or bunnies or pig or pigs or swine or porcine or equine or bovine or rodent* or feline or canine or sheep or lamb or lambs or cow or cows or calf or calves or kitten or puppy or puppies)	74

BASE: February 22, 2019

#	Search Statement	Results
1	tit:biomarker* and tit: hearing doctype:1*	12
2	Tit:connexin tit:inner tit:ear doctype:1*	15
3	Titexome tit:hearing doctype:1*	41
4	Tit:connexin tit:hearing doctype:1*	102
